# Inferring selection in the *Anopheles gambiae *species complex: an example from immune-related serine protease inhibitors

**DOI:** 10.1186/1475-2875-8-117

**Published:** 2009-06-04

**Authors:** Darren J Obbard, John J Welch, Tom J Little

**Affiliations:** 1 Institute of Evolutionary Biology, University of Edinburgh, Kings Buildings, West Mains Road, Edinburgh, UK

## Abstract

**Background:**

Mosquitoes of the *Anopheles gambiae *species complex are the primary vectors of human malaria in sub-Saharan Africa. Many host genes have been shown to affect *Plasmodium *development in the mosquito, and so are expected to engage in an evolutionary arms race with the pathogen. However, there is little conclusive evidence that any of these mosquito genes evolve rapidly, or show other signatures of adaptive evolution.

**Methods:**

Three serine protease inhibitors have previously been identified as candidate immune system genes mediating mosquito-Plasmodium interaction, and serine protease inhibitors have been identified as hot-spots of adaptive evolution in other taxa. Population-genetic tests for selection, including a recent multi-gene extension of the McDonald-Kreitman test, were applied to 16 serine protease inhibitors and 16 other genes sampled from the *An. gambiae *species complex in both East and West Africa.

**Results:**

Serine protease inhibitors were found to show a marginally significant trend towards higher levels of amino acid diversity than other genes, and display extensive genetic structuring associated with the 2La chromosomal inversion. However, although serpins are candidate targets for strong parasite-mediated selection, no evidence was found for rapid adaptive evolution in these genes.

**Conclusion:**

It is well known that phylogenetic and population history in the *An. gambiae *complex can present special problems for the application of standard population-genetic tests for selection, and this may explain the failure of this study to detect selection acting on serine protease inhibitors. The pitfalls of uncritically applying these tests in this species complex are highlighted, and the future prospects for detecting selection acting on the *An. gambiae *genome are discussed.

## Background

By vectoring *Plasmodium *parasites, *Anopheles *mosquitoes are a central component of the Malaria crisis. Consequently, there has been a substantial effort to identify the genes involved in the mosquito immune response against *Plasmodium*, including studies to identify genes associated with variation in vector competence [1-4]. It has been widely hypothesized that these immune response genes may be subject to strong parasite-mediated selection, such as that which occurs in a coevolutionary 'arms-race' [5,6]. Such arms-races involve strong reciprocally-antagonistic selection, leading to the frequent and rapid fixation of new alleles. This reduces within-species diversity, while driving between-species protein divergence, and leaves a genomic signature of past selection that can be identified through DNA sequence analysis [7,8]. Thus, DNA sequence analysis and the tools of population genetics can augment understanding of immune gene function in host-parasite interaction by identifying genes that are the target of parasite adaptation, and even distinguish between forms of parasite-mediated selection [5,6,9].

Population genetic methods have previously shed light on the nature and intensity of selection in both mammalian and *Drosophila *immune systems. For example, *Drosophila *studies have suggested that pathogens which manipulate signal transduction pathways or the antiviral RNAi pathway have been a major selective force [10,11]. In *Anopheles *mosquitoes, the potential for immune-related genes to determine vector competence provides a clear incentive to elucidate the selective forces that drive evolution. Serine protease inhibitors (serpins, or SRPNs) are prime candidates for such parasite-mediated selection in *Anopheles *mosquitoes. Serpins comprise a large and rapidly evolving super-family of proteins (reviewed in [12,13]) with key roles in the immune systems of vertebrates [14] and invertebrates [15]. In particular, *Drosophila *serpins, such as Nec and SRPN27A, modulate two of the most important defense pathways: the Toll-pathway [16,17], and the melanization cascade [18,19], and many are up-regulated on septic injury (Spn28D, SRPN27A, Spn5, CG6687 and Spn4, see [20]). Moreover, some *Drosophila *serpins display very high rates of amino acid substitution, and/or other signatures of adaptive evolution, e.g. [21-23].

Three *Anopheles *serpins have been experimentally associated with *Plasmodium*-interaction phenotypes (see Table 1). In *Anopheles gambiae *and *Anopheles stephensi *SRPN10 is expressed in the mosquito midgut and in haemocytes [24], and during *Plasmodium berghei *(a rodent parasite) invasion of the midgut epithelium SRPN10 moves from the nucleus to the cytoplasm, and its expression is strongly induced [25]. SRPN6 is also expressed in infected midgut epithelial cells and in haemocytes, and again its expression is strongly induced by *P. berghei *invasion in both *An. gambiae *and *An. stephensi*. The expression of SRPN6 is also induced by the human parasite *Plasmodium falciparum *[26]. RNAi knockdown of SRPN6 in *An. stephensi *resulted in a significant increase in the number of developing *P. berghei *oocysts, and although knockdown had no effect on oocyst numbers in susceptible strains of *An. gambiae*, in a resistant strain, the number of melanized *P. berghei *ookinetes was significantly increased [26]. More recently it has also been shown that SRPN6 is induced in the salivary glands of *An. gambiae *in response to *P. berghei *sporozoite invasion, and knock-down of SRPN6 by RNAi significantly increases the number of sprozoites reaching the salivary glands [27]. Finally, knockdown of SRPN2 in *P. berghei*-susceptible *An. gambiae *has a broadly opposite effect, resulting in a 97% decrease in oocyst formation through increased lysis and melanization, following midgut invasion [28].

**Table 1:** Locus Details and location

NAME	Identifier	Putative function	Genomic location
SRPN1	AGAP006909	Inhibitory Serine Protease inhibitor	2L:39892128-39893864
SRPN2	AGAP006911	Plasmodium-related Inhibitory Serine Protease inhibitor	2L:39897002-39899744
SRPN3	AGAP006910	Inhibitory Serine Protease inhibitor	2L:39895229-39896338
SRPN4C	AGAP009670	Inhibitory Serine Protease inhibitor	3R:38145527-38154288
SRPN5	AGAP009221	Inhibitory Serine Protease inhibitor	3R:28858000-28859778
SRPN6	AGAP009212	Plasmodium-related Inhibitory Serine Protease inhibitor	3R:28811997-28818217
SRPN7	AGAP007693	Inhibitory Serine Protease inhibitor	2L:49090665-49091915
SRPN8	AGAP003194	Inhibitory Serine Protease inhibitor	2R:33744972-33746720
SRPN9	AGAP003139	Inhibitory Serine Protease inhibitor	2R:33148444-33154607
SRPN10	AGAP005246	Plasmodium-related Inhibitory Serine Protease inhibitor	2L:12996143-13001508
SRPN11	AGAP001377	Non-inhibitory Serine Protease inhibitor	2R:4017728-4019706
SRPN12	AGAP001375	Non-inhibitory Serine Protease inhibitor	2R:4010431-4012512
SRPN14	AGAP007692	Non-inhibitory Serine Protease inhibitor	2L:49084812-49086463
SRPN16	AGAP009213	Inhibitory Serine Protease inhibitor	3R:28824548-28826209
SRPN17	AGAP001376	Inhibitory Serine Protease inhibitor	2R:4015617-4016537
SRPN18	AGAP007691	Non-inhibitory Serine Protease inhibitor	2L:49086842-49088278

Control1	AGAP006906	Adenosine deaminase-related growth factor	2L:39852471-39854636
Control2	AGAP006904	Matrix metalloproteinase	2L:39831595-39836700
Control3	AGAP006918	Putative NADH:ubiquinone dehydrogenase	2L:39995907-39997095
Control4	AGAP009673	glutaminyl-peptide cyclotransferase	3R:38248845-38249780
Control5	ENSANGG8091	(retrotransposon)	3R:28965847-28968579
Control6	AGAP009207	Mitogen-activated protein kinase ERK	3R:28697030-28708787
Control7	AGAP007712	Putative RHO guanyl-nucleotide exchange factor	2L:49181235-49190516
Control8	AGAP003205	Similar to Drosophila CG8468	2R:33825401-33827998
Control9	AGAP003143	Similar to Drosophila CG9904	2R:33211906-33213476
Control10	AGAP005247	no annotation	2L:13062962-13067750
Control11	AGAP001384	cAMP-dependent protein kinase, beta-catalytic subunit	2R:4098545-4103634
Control12	AGAP001371	Similar to Drosophila CG18643	2R:3885127-3885956
Control14	AGAP007713	Similar to human solute carrier family 39	2L:49196817-49198177
Control16	AGAP900209	DNA-directed RNA polymerase II subunit J	3R:28746535-28747425
Control17	AGAP001388	Similar to human mab-3-related transcription factor 3	2R:4120810-4122586
Control18	AGAP007717	Similar to Drosophila CAP CG18408-PE	2L:49212258-49224889

Here, population-genetic approaches are used to search for evidence of natural selection acting on 16 serpin genes in the *An. gambiae *species complex, including those implicated in immune function. First, by comparing serpins to other nearby genes, patterns of genetic diversity within and between populations of *An. gambiae*, *Anopheles arabiensis*, and *Anopheles melas *are used to identify loci that deviate strongly from neutral predictions. Second, a recent extension of the McDonald-Kreitman test is used to test for evidence of adaptive substitution between species [29,30]. The data are then discussed in terms of on-going population processes in the *An. gambiae *complex, many of which have important implications for the robust inference of selection.

Serpins were found to have slightly higher levels of amino acid diversity than other genes, consistent with either reduced constraint, or potentially with balancing selection. In common with previous analyses. [31], considerable structuring of genetic diversity in the SRPN1-2-3 cluster was found in association with the 2La chromosomal inversion. However, although serpins are good *a priori *candidates as targets for strong 'arms-race' selection, as with similar studies on other *Anopheles gambiae *immune-related genes (e.g. [6,32-34]), the tests for adaptive evolution presented here are largely inconclusive. The results show how standard population-genetic tests for selection may be difficult to apply in the *An. gambiae *species complex; this is due to for both demographic and phylogenetic factors that are already widely known, and further supported by the present data.

## Methods

### Samples

*Anopheles gambiae *individuals were collected from West Africa ('BK': Burkina Faso, Koubri village, 12°11'54 N; 1°23'43W) and East Africa ('KY': Kenya, Mbita, Suba District). *Anopheles arabiensis *individuals were also collected from West Africa ('BK', in the same collections as *An. gambiae*, above) and East Africa ('TZ', Tanzania, Ifakara). All *An. gambiae *and *An. arabiensis *used in this study were provided by H. M. Ferguson (University of Glasgow, UK). *Anopheles melas *individuals were collected from Coastal Ghana (Ghana, Essiama, 4°57.4 N; 2°24.1W) by N. Tuno (Institute of Tropical Medicine, Nagasaki University, Japan). *Anopheles merus *and *Anopheles quadriannulatus *species *A *(hereafter *An. quadriannulatus*) were both obtained from laboratory colonies, maintained by the Medical Research Council of South Africa (provided by R. Maharaj; MRC, Durban, South Africa) and the University of Wageningen (Strain 'Sangqua', Zimbabwe, provided by W. Takken), respectively.

The species identity of all *gambiae *complex members was verified by diagnostic PCR [35], and the *M *and *S *molecular forms of *An. gambiae *were distinguished by PCR-RFLP [e.g. [36]]. As expected from their known geographic distributions [37], all KY *An. gambiae *individuals were *S *form, and all but two of the BK *An. gambiae *sample were *M*-form. All *An. gambiae *individuals were also surveyed for 2La/+ chromosomal inversion status, using the PCR assay of White et al [38], derived from the sequenced breakpoints [39]. As reported previously, in addition to the expected diagnostic 207 bp and 492 bp fragment lengths, these primers were found to amplify fragments of lengths *ca*. 687 bp, 672 bp, 760 bp and 1020 bp in some individuals [32]. Direct sequencing of these fragments from a subset of individuals suggest they are insertion/deletion derivatives of expected assay products [32,40], and within the polymorphic KY population the 2La/2L+^a ^amplification fragments were in Hardy-Weinberg equilibrium (51 individuals, χ^2 ^= 0.44, 1df., *p *= 0.51), allowing us to tentatively assign 2La/2L+^a ^inversion status to all individuals [32].

### Loci

Thirty-two loci were selected for sequencing and analysis, including 16 of the 18 serpins currently identified in the *An. gambiae *genome (M. Kanost and K. Michel, pers. comm.), and 16 other protein-coding loci chosen to match the genomic position of the serpins without regard to function. SRPN19 (a non-inhibitory serpin with 1:1:1 orthologs in *Drosophila melanogaster*, *Aedes aegypti *and *An. gambiae*) and SRPN13, which does not appear in the current *An. gambiae *assembly, were not sequenced. The total sequenced length was ~19 Kbp of coding sequence per individual (i.e. approximately 600 bp from each locus; range 240 bp–800 bp). Not all loci were sequenced from the same individuals within populations, and not all loci were amplified from *An. melas*. A full summary of gene names, locations, and classification is presented in Table 1.

The 'control' loci should represent an unbiased sample of *Anopheles *genes, to which serpins can be compared. Because these control genes are position-matched, each lying ~90 Kbp (range 40–125 Kbp) from a 'partner' serpin, they should control for the effects of large-scale position-based variation in recombination and mutation rates. Note that improvements to the *An. gambiae *annotation have subsequently identified control locus 5 (previously annotated as ENSANGG000008091) as deriving from a retrotransposon (AgamP3.4, July, 2007).

### PCR and sequencing

Genomic DNA was extracted from single mosquitoes using DNeasy kits (QIAgen). PCR primers were designed from the published *An. gambiae *genome sequence [41], and the final primer sequences selected after trouble-shooting (sequences are given in Additional file 1). Only one PCR amplicon was used per locus, thus sequences do not represent entire genes. Following PCR, unincorporated primers and dNTPs were removed using exonuclease I (New England BioLabs) and shrimp alkaline phosphatase (Amersham). PCR products were sequenced in both directions using BigDye™ reagents (v3.1, Applied BioSystems) and an ABI capillary sequencer. In some amplicons, indel polymorphism required the use of additional sequencing primers. The sequence chromatograms were assembled using SeqManII (DNAstar Inc., Madison USA) then inspected by eye to confirm the validity of all differences within and between species and all heterozygous base-calls. The heterozygous sequence from each diploid individual was decomposed into two pseudohaplotypes for analysis using PHASE [42,43]. However, the presented analyses should be highly robust to any errors in phase assignment, as only explicitly tree-based results, such as Hudson's nearest neighbour statistic (Snn), are affected by allelic phase. All unphased sequences have been submitted to GenBank as population sets, using ambiguity codes to indicate heterozygous sites. Sequence accession numbers span the range GQ146469–GQ148534.

### Divergence, diversity and differentiation

The number of synonymous and non-synonymous polymorphisms and substitutions, and the average pairwise genetic diversity (*π*) were calculated using DnaSP [version 4.50.3, ref [44]]. Diversity was calculated separately for synonymous (*π*_*s*_) and non-synonymous (*π*_*a*_) sites, and used a Jukes-Cantor correction for multiple substitutions, as implemented in DnaSP. Departures from the allele frequency spectrum expected under the standard neutral model were quantified using Tajima's *D *statistic [45], also calculated using DnaSP. Tajima's *D *(which measures departures from the expected allele frequency distribution under a standard neutral model) was calculated using synonymous sites only, and was calculated separately for both populations of *An. gambiae *and *An. arabiensis *(but not for *An. melas*, where sample sizes were too small to give meaningful results). The significance of departures from the expected allele frequency distribution were tested using 10,000 rounds of coalescent simulation (as implemented in DnaSP) conditional on the number of segregating sites and conservatively assuming no recombination within loci.

Genetic differentiation between populations was quantified in DnaSP using Hudson's *K*_*ST *_statistic [equations 7 to 9 in reference 46] which is calculated from the average number of pairwise differences between sequences taken within populations and between all populations, and is identical to Nei's *γ*_*ST *_[47] except for the population-size weighting scheme [see [46]]. Significant departures from zero population differentiation were inferred by permuting sequences between populations to create a null distribution of *K*_*ST *_values. All non-parametric statistical tests on diversity and differentiation were performed using the R statistical language (R Development Core Team, 2008 ). Although non-parametric tests (Spearman's rank correlation, paired Wilcoxon tests) are presented below, except where noted explicitly, parametric equivalents (Pearson's correlation, paired t-tests) gave qualitatively identical results.

### The proportion of adaptive substitutions

If it is assumed that synonymous mutations are effectively neutral, and that the fixation or loss of selected amino-acid variants is so rapid that the vast majority of non-synonymous polymorphisms are also effectively neutral, then the relative numbers of polymorphisms (*P*, within species) and fixed differences (*D*, between species) at synonymous and non-synonymous sites can be used to identify the action of selection [see [7] for an introduction]. This forms the basis of the McDonald-Kreitman test [MK test, [29]], which seeks to detect a departure from independence in a simple 2 × 2 contingency table of polymorphisms (*P*_*N *_and *P*_*S*_) and fixed differences (*D*_*N *_and *D*_*S*_). For a single gene, the departure from neutrality can easily be quantified by summary statistic such as the neutrality index (N.I.= (*P*_*N*_/*P*_*S*_)/(*D*_*N*_/*D*_*S*_) [48]), or by the estimated proportion of adaptive substitutions (α = 1 - (*D*_*S*_*P*_*N*_)/(*D*_*N*_*P*_*S*_) = 1 - N.I., [49]). This approach can be extended to multiple genes using *D*_*S*_, *P*_*N*_, *D*_*N *_and *P*_*S *_averaged across genes [49], or using a more sophisticated maximum-likelihood estimator of *α*, such as that of Bierne and Eyre-Walker [50].

Here, an extension of the maximum-likelihood method of Welch [30] was used. This method is very closely related to that of Bierne and Eyre-Walker [50], but additionally allows for the possibility that some apparent fixed differences may actually be polymorphisms that only appear fixed due to small sample size [51]. The method was extended to include polymorphism values from two species simultaneously e.g. [29,51]. Using this approach, models were fitted in which expected neutral divergence, λ = μ*t*, took a single value at all loci; expected neutral diversity, θ = 4*N*_*e*_μ, was also shared between all loci, but free to vary between species; and selective constraint, *f*, was free to vary between loci [see [30] for other details of the model]. Three nested models were fitted, in which (1) α was constrained to zero at all loci, i.e. no adaptive evolution, (2) a single α was shared by all genes, and (3) α was free to differ between serpins and 'control' genes. In this way, it was possible to test both for evidence of adaptive evolution, and whether serpins have a different rate of adaptive evolution to other genes. Model fit was tested using both likelihood ratio tests and Akaike weighting (derived from the Akaike information criterion) [52]. Confidence intervals on α were obtained by adjusting α away from its maximum likelihood value, and allowing the other parameters to take their maximum likelihood value, conditional on that α, until log likelihood decreased by 2 units [50]. C code to fit these models is available on request from the authors, or from [53].

## Results

### Synonymous site diversity

**Figure 1:**
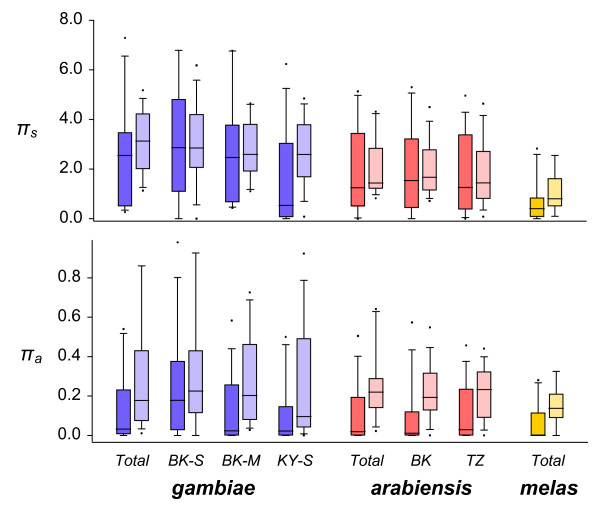
**Genetic diversity at synonymous and non-synonymous sites**. Genetic diversity (the percentage of sites that differ on average between haplotypes) at synonymous (*π*_*s*_) and nonsynonymous (*π*_*a*_) sites measured at 32 loci in populations of *An. gambiae*, *An. arabiensis *and *An. melas*. Diversity is shown separately for control loci (dark bars) and serpins (pale bars), and is shown for the species as a whole, and for each population separately. Note that although only two individuals (4 haplotypes) were sampled for S-form *An. gambiae *in population BK, ~19 Kbp of sequence will provide a good estimate of *π *if mating within the population is random. Diversity was significantly higher in *An. gambiae *than in *An. arabiensis*, and significantly lower in *An. melas*. For non-synonymous sites, serpins had significantly higher diversity than control loci, but this trend was non-significant at synonymous sites. See main text for details, and Additional File 1 for the raw data.

Across all 32 loci, average pairwise genetic diversity at synonymous sites (*π*_*s*_) was highest in *An. gambiae *(*π*_*s *_= 2.82%, 95% bootstrap interval 2.25–3.41%), lowest in *An. melas *(*π*_*s *_= 0.86%, 0.53–1.19%), and intermediate in *An. arabiensis *(*π*_*s *_= 1.97%, [1.50, 2.46]; Figure 1a). The difference in *π*_*s *_between *An. gambiae *and *An. arabiensis *was highly significant (Paired Wilcox test using 32 loci, V = 444, *p *= 0.0004), and *π*_*s *_correlated strongly between these species (Spearman's rho = 0.72, S = 1504, *p *< 6 × 10^-6^, Additional file 2). Diversity in *An. gambiae *and *An. arabiensis *did not correlate with diversity in *An. melas *(*p *> 0.5 in both cases, 26 loci). For a full summary of synonymous diversity, and all other summary stats that follow, see Additional file 3.

For *An. gambiae*, genetic diversity was slightly higher in West Africa (*π*_*s *_= 2.74%; BK M-form) than in East Africa (*π*_*s *_= 2.17%; KY S-form), and although the effect was small, it was statistically significant (Paired Wilcox V = 380, *p *= 0.03, Figure 1a). Diversity was also highly correlated between East- and West-African populations (Spearman's rho = 0.72, S = 1520, *p *= 3 × 10^-6^). Although only two S-form individuals were sampled from West Africa, they displayed higher diversity than the either of the other two *An. gambiae *populations (BK S-form; *π*_*s *_= 2.98%). For *An. arabiensis*, diversity correlated even more strongly between East and West Africa (rho = 0.87, S = 729, *p *< 2 × 10^-10^) and did not differ significantly between the populations (*π*_*s *_= 1.96% in BK vs. *π*_*s *_= 1.79% in TZ, Paired Wilcoxon test V = 316, *p *= 0.19).

Synonymous site diversity did not differ significantly between serpins and other genes in either *An. gambiae *(3.10% vs. 2.53%, Paired Wilcoxon V = 48, *p *= 0.32, Figure 1a) or *An. arabiensis *(2.00% vs. 1.93%, Paired Wilcox V = 60, *p *= 0.71). Although position-matched, no correlation in diversity could be detected between serpins and their corresponding control genes (*p *> 0.1 in both cases), suggesting that the effect of genomic location on neutral diversity was relatively weak.

### Non-synonymous site diversity

Non-synonymous diversity (*π*_*a*_) was very similar between *An. gambiae *(π_*a *_= 0.22%; 95% bootstrap interval 0.13–0.31%) and *An. arabiensis *(*π*_*a *_= 0.18%; bootstrap interval: 0.12–0.24%, Figure 1b), and did not differ significantly between the species (Paired Wilcoxon test V = 285, *p *= 0.29). Although *π*_*a *_did not correlate significantly with *π*_*s *_in either *An. gambiae *or *An. arabiensis *(correlation coefficients were 0.17 and 0.07 respectively, *p *> 0.3 in both cases), there was a strong correlation in *π*_*a *_between the two species (rho = 0.81, S = 1037, *p *= 2 × 10^-8^, Additional file 2). East and West African populations did not differ significantly in *π*_*a *_for either *An. gambiae *or *An. arabiensis *(*p *> 0.1 in both cases), but *π*_*a *_did correlate very highly between East and West African populations (rho = 0.80 *p *= 6. × 10^-8^, and rho = 0.88, p = 2 × 10^-11^, respectively). Interestingly, π_*a *_was higher for serpins than for other genes in both *An. gambiae *and *An. arabiensis *(*π*_*a *_= 0.30% vs. 0.13%, and *π*_*a *_= 0.25% vs. 0.11%), although statistical significance was marginal for *An. gambiae *(Paired Wilcoxon V = 30 *p *= 0.051, and V = 24, *p *= 0.024 for *An. gambiae *and *An. arabiensis *respectively, Figure 1b). Despite fewer loci being sequenced, this effect could also be detected in *An. melas *(π_a _= 0.15% vs. 0.06%, unpaired Wilcoxon test W = 25, *p *= 0.034, Figure 1b).

### Allele frequency spectra

Tajima's *D *statistic for synonymous sites was negative in both populations of *An. arabiensis *(*D *= -0.29 and *D *= -0.38, averages across loci in TZ and BK respectively) and in *An. gambiae *BK (*D *= -0.71, *M*-form individuals only), and did not differ between serpins and other genes (Wilcoxon tests, *p *> 0.5 in all cases). Tajima's *D *did not differ significantly between the two populations of *An. arabiensis *(Paired Wilcoxon V = 193, *p *= 0.42), but did correlate between the populations (rho = 0.43 S = 2543.131, *p *= 0.017). In population BK, Tajima's *D *was correlated between *An. gambiae *and *An. arabiensis *(rho = 0.490 S = 2293, *p *= 0.006), but was significantly more negative in *An. gambiae *(Paired Wilcoxon V = 115, *p *= 0.015). Strikingly, Tajima's *D *was generally positive in *An. gambiae *population KY (mean across loci 0.77, 22 out of the 28 genes with non-zero diversity had *D *> 0, with overall 95% bootstrap interval [0.40, 1.15]). This was significantly higher than in BK (Paired Wilcoxon test V = 15, *p *= 1 × 10^-6^).

In *An. arabiensis*, five genes had individually significantly negative Tajima's D statistics (*p *< 0.05 in all cases, no correction for multiple tests): control loci 1 (BK and TZ) and 5 (BK), and serpins 10 (BK), 6 and 7 (TZ). In *An. gambiae *population BK (*M*-form only) 7 genes had significantly negative *D *values: serpins 7, 9 and 14, and control loci 5, 6, 10 and 11 (*p *< 0.05 in all cases).

### Genetic differentiation between populations

**Figure 2:**
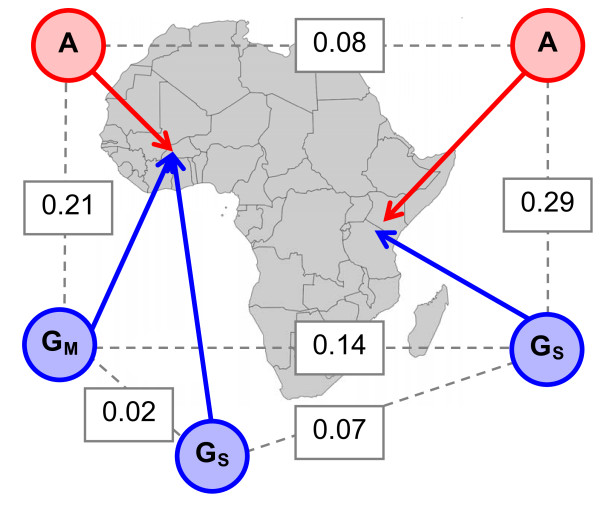
**Genetic differentiation between populations**. Arrows indicate approximate sampling locations within Africa, and letters identify species (A- *An. arabiensis*, G- *An. gambiae*, M-form and S-form). Dashed lines indicate pairs of populations for which genetic differentiation was calculated, and numbers are *K*_*ST *_statistics, averaged across all 32 loci.

In *An. arabiensis*, differentiation between East and West Africa was very low (*K*_*ST *_= 0.08) and not significantly different from zero at 15 of the 32 loci examined. In *An. gambiae*, differentiation between East and West Africa was much higher (*K*_*ST *_= 0.14; all loci except control locus 9 were individually significantly differentiated) and this difference between the species was statistically significant (paired Wilcoxon test V = 392, *p *= 0.016). Note that this differentiation reflects not only geographic separation, but also differentiation between *M *and *S *molecular forms of *An. gambiae*. In West Africa (BK) differentiation between *M*- and *S-*form was very low (*K*_*ST *_= 0.016), and significantly lower than differentiation between *S*-form sampled from East Africa and *S*-form sampled from West Africa (*K*_*ST *_= 0.073, paired Wilcoxon test V = 442, *p *= 0.0005). Only two S-form *An. gambiae *individuals were sampled from BK, making estimates of differentiation potentially poor and reducing the power of the test. However, assuming random mating, the estimates should not be biased by the small number of individuals sampled, and the large number of loci (32, ~19 Kbp of sequence) will reduce sampling error. For an overview of genetic differentiation see Figure 2.

### The 2La chromosomal inversion

*Anopheles gambiae *population KY was highly polymorphic for the 2La/+ chromosomal inversion, allowing us to test for differentiation between the two inversion states. Dividing population KY into two groups on the basis of on inversion status (2La homozygotes *versus *2L+^a ^homozygotes, heterozygotes excluded) identified very strong population structure associated with the inversion. At the six loci sampled from within the inversion (serpins 1–3 and control loci 1–3) mean differentiation between inversion-groups was *K*_*ST *_= 0.25 (all six loci were significantly differentiated: permutation *p *< 0.01 for each locus). Across the six other polymorphic loci sequenced from chromosome arm 2L (serpins 7, 10, 14 and 18, control loci 10 and 18), differentiation between these groupings was *K*_*ST *_= 0.03, and was not significantly different from zero in any locus (*p *> 0.14 by permutation, in each locus).

### Divergence and differentiation between species

**Figure 3:**
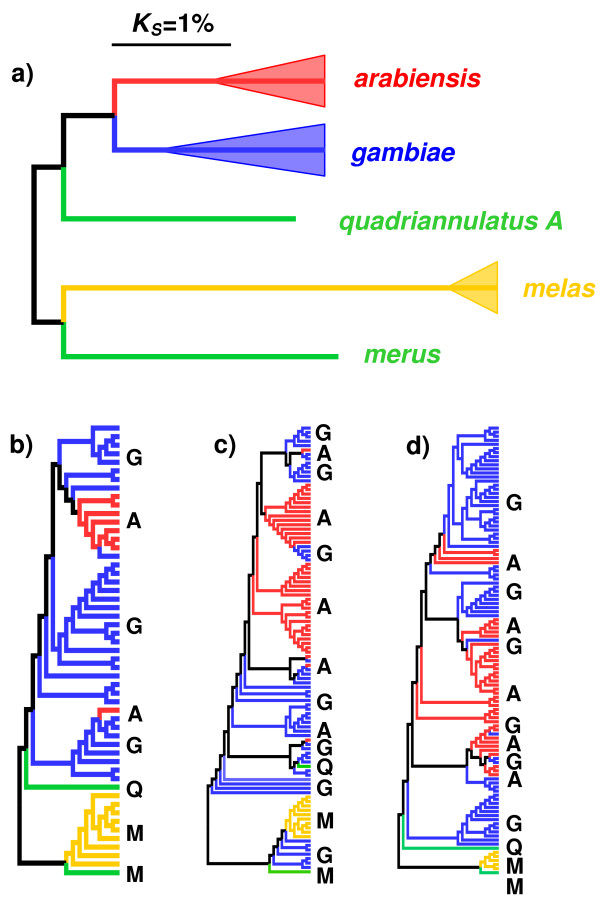
**Genetic divergence within the *gambiae *species complex**. (a) An un-rooted neighbour-joining tree, calculated from pairwise *K*_*S *_between species averaged across loci. Branch lengths are to scale. The filled triangles illustrate the relative scale of diversity and divergence within the complex, such that the length of the triangle is half the divergence between haplotypes within species (i.e *π*_*s*_/2) and net divergence (*K*_*S*_-*π*_*s*_) corresponds to branch-lengths that are not part of the triangle. (Note that population samples and thus *π*_*s *_were not available for *An. quadriannulatus A *and *An. merus*). (b)-(d) Neighbour-joining cladograms (i.e. topology only, branch-lengths uninformative) showing the unique alleles sequenced from three loci. Note that in all cases *An. gambiae *and *An. arabiensis *alleles are intermixed. (b) to (d) are control locus 14, SRPN7 and SRPN11, selected to illustrate a wide range of *K*_*ST *_values between *An. gambiae *and *An. arabiensis *(*K*_*ST *_= 0.51, 0.10 and 0.09, respectively).

Genetic divergence (substitutions per site) between *An. gambiae *and *An. arabiensis *was very low: *K*_*S *_= 3.5% (averaged across loci), and when corrected for diversity (i.e. , page 220 in [54]), *K*_*S *_= 1.2%. No synonymous fixed differences were identified between these species, and only three non-synonymous fixed differences (all in control locus 5, derived from a transposable element). Divergence from *An. melas *was higher for both *An. gambiae *(*K*_*S *_= 6.4%, corrected *K*_*S *_= 4.6%) and *An. arabiensis *(*K*_*S *_= 6.3%, corrected *K*_*S *_= 4.9%), as was divergence from *An. merus *(uncorrected *K*_*S *_= 5.3% and *K*_*S *_= 4.8%, respectively); divergence between these species and *An. quadriannulatus *was intermediate (uncorrected): *K*_*S *_= 4.01% and *K*_*S *_= 4.30% respectively. For an overview of interspecies divergence and an illustration of gene trees versus species trees, see Figure 3.

Genetic differentiation (the proportion of total diversity attributable to between-species differences) between *An. gambiae *and *An. arabiensis *species was *K*_*ST *_= 0.19 (95% bootstrap interval across loci [0.16, 0.24]). Differentiation between *An. gambiae *and *An. melas *was much higher (*K*_*ST *_= 0.33 [0.27, 0.40]), as was differentiation between *An. arabiensis *and *An. melas *(*K*_*ST *_= 0.50 [0.44, 0.57]). Differentiation between *An. gambiae *and *An. arabiensis *was lower for serpins than for control loci (*K*_*ST *_= 0.14 vs. *K*_*ST *_= 0.25, paired Wilcox test V = 113, *p *= 0.02). However, although the trend was in the same direction in East and West Africa, this effect was only significant in West Africa (V = 108, *p *= 0.04, as compared to V = 99, *p *= 0.12).

### Inference of adaptive substitutions

Due to the lack of fixed differences between *An. gambiae*, *An. arabiensis *and *An. quadriannulatus*, McDonald-Kreitman based approaches were not applied to these data (see Discussion). However, it was possible to apply the method of Welch [30] using *An. gambiae*/*An. arabiensis*, and their divergence from *An. melas *and/or *An. merus*. According to the likelihood ratio test, neither analysis using *An. melas *provided any evidence supporting adaptive substitutions between the lineages (there was no significant improvement in model fit between fixing α = 0 and allowing α to take its maximum likelihood value; *p *> 0.19 in all cases: Table 2). This was also true for the test using *An. gambiae *and *An. merus *(*p *> 0.2: Table 2). For the test using *An. arabiensis *and *An. merus *there was some evidence that α was significantly greater than zero, (α = 0.34 [0.08, 0.53]; 2ΔlnL = 6.16, 1d.f., *p *= 0.013, significance lost if a correction is made for multiple tests), but no significant improvement in model fit was obtained by allowing α to differ between serpins and control loci, or between Plasmodium-related serpins and all other loci. This suggests that approximately 8–53% of amino-acid substitutions between *An. arabiensis *and *An. merus *were adaptive, but this value did not differ significantly between serpins and the control loci. Analysis of Akaike weights gives a qualitatively identical result: in each species-pair no model is strongly preferred, but in the *merus*-*arabiensis *comparison, α = 0 receives relatively little weight. Full results of the McDonald-Kreitman analysis are presented in table 2, and raw data are given in Additional file 4.

**Table 2:** Estimates of the proportion of adaptive substitutions

**α model**	**Par**	**log*(L)***	**2Δlog*(L)***	**χ^2 ^*p*-value**	**AIC**	**Akaike weight**	**α_a_**	**α_b_**
*An. gambiae vs. An. melas*								
α = 0	29	-336.49			730.98	**0.424**	[0]	[0]
α ~ (all loci)	30	-335.76	1.46	0.23	731.52	0.323	0.23	[0.23]
α ~ (control, serpin)	31	-335.66	0.19	0.91	733.33	0.131	0.18	0.25
α ~ (other, immune)	31	-335.73	0.06	0.97	733.46	0.123	0.24	0.07

*An. arabiensis vs. An. melas*								
α = 0	29	-329.36			716.73	**0.495**	[0]	[0]
α ~ (all loci)	30	-329.31	0.10	0.75	718.63	0.191	-0.05	[-0.05]
α ~ (control, serpin)	31	-328.07	2.48	0.29	718.15	0.243	-0.54	0.10
α ~ (other, immune)	31	-329.30	0.02	0.99	720.61	0.071	-0.03	-0.15

*An. gambiae vs. An. merus*								
α = 0	34	-333.36			734.72	**0.349**	[0]	[0]
α ~ (all loci)	35	-332.60	1.52	0.22	735.21	0.274	0.15	[0.15]
α ~ (control, serpin)	36	-331.59	2.02	0.36	735.18	0.277	0.34	0.03
α ~ (other, immune)	36	-332.60	0.00	1.00	737.21	0.101	0.15	0.17

*An. arabiensis vs. An. merus*								
α = 0	34	-311.04			690.07	0.053	[0]	[0]
α ~ (all loci)	35	-307.96	6.15	0.01	685.92	**0.422**	0.34	[0.34]
α ~ (control, serpin)	36	-307.22	1.48	0.48	686.44	0.325	0.47	0.25
α ~ (other, immune)	36	-307.71	0.50	0.78	687.42	0.200	0.36	0.10

α_a _and α_b _are estimates of the proportion of adaptive substitutions in each of the two classes of gene (control/serpin or non-immune/immune, respectively), Par is the number of parameters in the model. Where the value of α is constrained by the model it is marked in square brackets. Negative values arise from an 'excess' of non-synonymous polymorphism, and could represent sampling error, or mildly deleterious polymorphisms [7]. AIC is the Akaike Information Criterion. The Akaike weighting can be interpreted as the weight of evidence in favour of the corresponding model, given the relative support for all the available models [52].

## Discussion

### Population history and speciation in Anopheles

The *An. gambiae *complex falls within the *Pyretophorus *series of the subgenus *Cellia*, and comprises a closely-related group of approximately eight species (*An. gambiae s.s., An. arabiensis, Anopheles bwambae, An. quadriannulatus A and B, An. merus, An. melas and Anopheles comorensis*) [55-57], plus at least one case of incipient speciation (*M *and *S *molecular forms of *An. gambiae *s.s. [37,58-60]). Because lineages within the complex differ in their importance as *Plasmodium *vectors [e.g., [61]], in their ecological preferences [62,63], and in their resistance to pesticides [e.g. [63]], there is considerable value in understanding both species relationships and how populations are structured. This may be of particular consequence if any attempt is ever made to genetically modify wild *Anopheles *populations to block or reduce *Plasmodium *transmission [64,65]. However, in addition to having important implications for vector control, as discussed below, understanding phylogeny and population history are also essential to the robust inference of selection.

Previous analyses suggest that *An. gambiae *and *An. arabiensis *are sister taxa, and the data presented here from five of the eight species are in strong agreement, placing *An. gambiae *and *An. arabiensis *as the most closely related species pair, with greater divergence to *An. merus *and *An. melas *(Figure 3a; note that the tree is unrooted). However, in common with previous studies [e.g. [33,66,67]], these data suggest extensive shared polymorphism (Figure 3) and very low differentiation between *An. gambiae *and *An. arabiensis *(*K*_*ST *_= 0.19). The inter-species differentiation between *An. gambiae *and *An. arabiensis *is approximately the same as inter-population differentiation between African and European *D. melanogaster *(*K*_*ST *_= 0.16 to 0.24, depending on population; pers. comm. P. R. Haddrill, data from [68]), and is lower than inter-population differentiation in the predominantly selfing nematode *Caenorhabditis elegans *(*K*_*ST *_= 0.38, pers. com. A. D. Cutter, data from [69]). Thus, although *An. gambiae *and *An. arabiensis *are largely reproductively isolated and significantly differentiated [70,71], these data confirm that either they share extensive ancestral polymorphism, or that there is considerable introgression between them [see also [67,72,73]]. This is further supported by the very high correlation in neutral diversity across genes, between these two species (Additional file 2).

Given a particular divergence time, effective population size is the primary determinant of the amount of shared ancestral polymorphism between taxa, because drift (and therefore lineage-sorting) is faster in small populations [e.g. [74]]. Thus, although it is likely that *An. gambiae *and *An. arabiensis *share a more recent common ancestor that either does with *An. melas*, the lower differentiation between *An. gambiae *and *An. arabiensis *could also be explained (at least in part) by differences in effective population size within the complex. Since *π*_*s *_is an estimator of 4*N*_*e*_μ, differences in neutral diversity imply that *An. gambiae *and *An. arabiensis *have larger effective population sizes than *An. merus*, consistent both with their wider geographic range, and with potentially higher levels of shared ancestral polymorphism (see above: *π*_*s*_~2.8%, 2.0%, and 0.9% for *An. gambiae*, *An. arabiensis *and *An. melas*, respectively). Diversity in *An. gambiae *and *An. arabiensis *is similar to that seen in African populations of *Drosophila simulans *and *D. melanogaster *(*π*_*s *_= 3.2% and *π*_*s *_= 1.7%, respectively) [e.g. [75]], and assuming the mutation rate (μ) is similar between mosquitoes and *Drosophila*, this also suggests a long-term effective population size for *An. gambiae *that is about 70% larger than *D. melanogaster *[76], i.e. well in excess of 1 million. This is broadly consistent with previous estimates from mitochondrial sequence, but is much larger than estimates based on microsatellites and allozyme variants [reviewed in [77]].

Incipient speciation between the *M *and *S *molecular forms of *An. gambiae *is a major focus of ongoing research [37,58-60], culminating in the recently completed sequencing of the *M *and *S *molecular-form genomes [78]. Although differentiation between the *M *and *S *form of *An. gambiae *in West Africa is extremely low (*K*_*ST *_= 0.02; Figure 2), it is consistently non-zero at some loci, even where the lineages are sympatric (see e.g. [6]). This unambiguously identifies *M *and *S *form *An. gambiae *as being (at least partly) reproductively isolated [58], and it has been argued that different *M *and *S*-form niches may be distinguishable [37]. Moreover, differentiation is variable around the genome, being higher at so-called 'islands of speciation', potentially associated with adaptive differences [58,59]. However, other studies have shown that in some geographic regions microclimate is a better predictor of population divergence than is molecular form [79], and it is clear that the S-form of *An. gambiae *is not a single homogenous lineage [66,80]. Indeed, it is well-established that there is extensive genetic differentiation associated with the Great Rift Valley [80], and consistent with this, the data presented here not only identify considerable differentiation between East and West Africa (KY S-form *versus *BK M-form, *K*_*ST *_= 0.12), but also show that West African *M*-form and *S*-form are less differentiated from each other than either is from East African S-form (Figure 2). This suggests either that the West African *M *and *S *lineages share a more recent common ancestor, or alternatively that they have experienced considerable recent gene flow [[37], e.g. [80], but see also [81]]. For *An. arabiensis*, differentiation across the width of the continent is only *K*_*ST *_= 0.08, which is very similar to that between Eastern and Western *S*-form *An. gambiae *(*K*_*ST *_= 0.07; Figure 2), and approximately twice that between *D. melanogaster *populations sampled across a similar geographic range (Gabon vs. Kenya or Zimbabwe, *K*_*ST *_= 0.04) [P. R. Haddrill pers. comm., data from [68]].

Most populations showed a slight skew in the allele-frequency spectrum toward low-frequency variants (i.e., average Tajima's *D *was negative), consistent either with population growth, or with weak selection against some synonymous variants. In contrast, however, the data presented here also identify a strong skew toward intermediate frequency alleles in *An. gambiae *population KY (i.e., a positive Tajima's *D*, 0.77 averaged across loci, 95% bootstrap interval 0.40–1.15). One potential explanation for this is that population KY is admixed or contains cryptic population structure, for example as would be the case if two divergent lineages of *S*-form are coexisting there. Alternatively, a positive Tajima's *D *could also result from a recent decrease in effective population size.

The phylogenetic and phylogeographic complexity of the *An. gambiae *species group means that inferences drawn from single individuals should be treated with caution, as the low differentiation between species and high diversity within species means that any one individual is not necessarily typical or representative [e.g. [66]]. For example, the recently sequenced *M *and *S*-form genomes [78] were both obtained from mosquitoes sampled in Mali, but *S*-form divergence between East and West Africa is considerably greater than *M*-*S *divergence within West Africa (see Figure 2 above, and [66,80], cf. [81]). It is therefore not clear that any conclusions regarding *M *- *S *genome divergence will generalize to S-form individuals from the east African coast. The same issue arises with inter-species comparisons. For example, the divergence between two randomly sampled *An. gambiae *genomes is *K*_*S*_~3%, and that between one randomly selected *An. gambiae *genome and one randomly selected *An. quadriannulatus *genome is only *K*_*S*_~4% (above, and Figure 3). This means that a single inbred strain of *An. quadriannulatus *is only marginally more informative about *An. quadriannulatus *than it is about *An. gambiae*, and without more extensive sampling it cannot reliably be used to identify genetic differences between the species [cf. [61]]. These issues will be of paramount importance in analysing the recently approved complete genomes of *An. arabiensis*, *An. quadriannulatus *and *An. merus *[82].

### Evidence for adaptive evolution in *Anopheles *serpins

There is considerable evidence from other taxa that serpins are an evolutionarily dynamic gene family, with high turnover between lineages and occasional lineage-specific expansions (e.g. [23], see [13] for a review). For example, although most *Anopheles *and *Aedes *serpins have 1:1 orthologs, there are 29 serpins in *D. melanogaster *but only 18 in *An. gambiae*, and very few mosquito serpins have 1:1 orthologs in *Drosophila *[83]. Some serpins also show very high rates of adaptive evolution, such as *Drosophila *Spn28D [CG7219 in ref. [21]], and it has been suggested that, in general, rapid turnover and strong selection in serpins may be associated with serpin immune function, and could be driven by an evolutionary 'arms-race' [13]. In *An. gambiae*, three serpins are known to have immune-related function in response to *Plasmodium *infection (SRPN2, SRPN6 and SRPN10 in [24,26,28]).

Strong selection can affect patterns of genetic diversity, both between populations, and between chromosomal inversions. In *An. gambiae *population KY, considerable differentiation was identified between 2La and 2L+^a ^homozygotes around the SRPN1, 2 and 3 cluster (and control loci 1, 2 and 3). For these loci *K*_*ST *_= 0.25 between inversion states, which is actually higher than the overall differentiation between *An. gambiae *and *An. arabiensis*, and twice as high as the differentiation between *M*-form and *S*-form *An. gambiae*. No such differentiation was seen for other loci on chromosome arm 2L, indicating that this is strongly associated with the inversion. Although chromosomal inversions are in general expected to suppress recombination, especially near breakpoints, this is unlikely to lead to extreme or long-term differentiation unless maintained by selection [e.g. [84,85]]. In particular, despite recombination being suppressed in heterozygous individuals, genetic exchange (including recombination and gene-conversion) within the region of the 2La/+ inversion is not zero [86], and this should allow such differentiation to break down rapidly if it is not selectively maintained. The finding of elevated differentiation around these loci agrees with previous analyses, which found the SRPN1-3 cluster to be close to the region of highest differentiation between 2La and 2L+^a^. [31]. While this doesn't provide strong evidence that any of these serpins are being directly selected, it is interesting to note that SRPN2 is required for successful infection of *An. gambiae *by *P. berghei *[28], and that 2La inversion-status was identified with vector-competence in some early studies [87,88]. Thus it is possible that genetic structuring in serpins 1–3, introduced and maintained by the 2La inversion, may affect variation in vector competence, even if the underlying cause of 2La/+ differentiation is elsewhere.

In contrast, using a McDonald-Kreitman based approach to detecting adaptive substitutions [29,30], no strong or consistent evidence of selection could be detected, nor could differences in the rate of adaptive evolution between serpins and other genes (or between immune serpins and other genes; Table 2). Specifically, in three of the four species pairings that were analysed, the rate of adaptive evolution could not be distinguished from zero (Table 2). This may indicate that neither *Anopheles *immune-related serpins, nor *Anopheles *serpins as a family, are subject to selection for rapid change, and consequently that all selection acting on these genes is purifying. However, it may also be an artefactual result arising either from limitations of the McDonald-Kreitman framework, or from issues specific to the gambiae species complex (see next section). In particular, the McDonald-Kreitman approach assumes that non-synonymous substitutions can be divided into three classes: strongly advantageous mutations that fix rapidly, strongly deleterious mutations that are rapidly lost, and effectively neutral mutations that drift in frequency [50]. If there is also a large class of weakly deleterious mutations that remain polymorphic for an extended period, but are lost by selection in the long term, then this will reduce estimates of α [50].

In agreement with this, a trend toward higher amino-acid diversity was found in serpins, as compared to other genes, in all three species (*An. gambiae*, *An. arabiensis *and *An. melas*; Figure 1b). This could suggest that purifying selection on serpins is weak or intermittent (as compared to purifying selection on other genes), or that there is selection favouring diversity in serpins, such as balancing selection [89]. However, if the latter were the case, then one might also expect to find an increase in diversity at linked synonymous sites, and although slightly higher, synonymous diversity in serpins did not differ significantly from other genes (Figure 1a), suggesting that they neither experience long-term selection for increased polymorphism, nor undergo more frequent selective sweeps [e.g. [8]]. Moreover, in no population or species did a serpin display the highest or lowest neutral genetic diversity, and there was no clear pattern in the allele frequency spectrum (as measured by Tajima's *D *statistic) or inter-population differentiation (measured by *K*_*ST*_) that supports the notion of strong selection acting on *Anopheles *serpins. These data therefore fail to identify any serpins as candidates for recent strong selection within the gambiae complex.

### Prospects and pitfalls in inferring adaptive evolution in *Anopheles*

A null result in tests for selection may also result from low power or model violations associated with the phylogenetic and population history in the *An. gambiae *complex. Motivated by an interest in identifying targets of pathogen-mediated selection, several studies have now attempted to identify adaptive evolution in immune-related genes from the *Anopheles gambiae *species complex [5,6,32-34,90-92]. However, in stark contrast to almost identical studies in taxa such as *Drosophila *[for example [10,11,21,93-95]], to date there is very little clear evidence supporting adaptive evolution in the *An. gambiae *complex [but see [90,92]].

**Figure 4:**
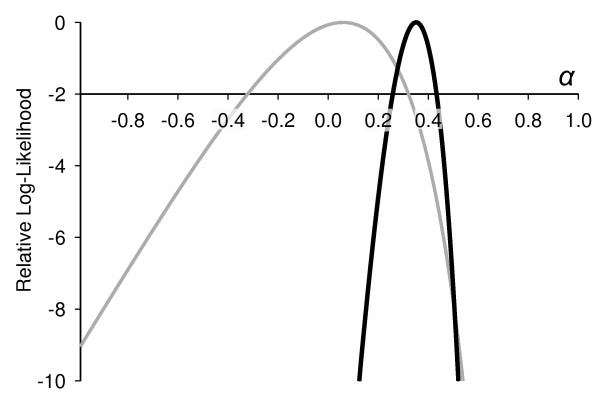
**The power to estimate α using *An. gambiae *and *An. arabiensis***. The relative log-likelihood of α (the proportion of amino-acid substitutions that are adaptive) estimated using the modified McDonald-Kreitman approach [30]. The grey curve is calculated from all 102 genes for which both *An. arabiensis *and *An. gambiae *population samples were available in the dataset of Cohuet et al. [6]. The black curve shows an equivalent dataset of 102 genes from *Drosophila melanogaster *and *D. simulans*, with genes selected to be the same average length as those in the Cohuet dataset (D. J. Obbard, J. J. Welch and F. M. Jiggins, unpublished data). Despite both pairs of species having similar levels of diversity (*π*_*s *_from 1.6% to 2.9%), for the *Anopheles *dataset the bounds (2 units of log Likelihood) stretch from -0.33 to 0.32 (and include zero) while for *Drosophila *the bounds only stretch from 0.26 to 0.44, and the maximum-likelihood estimate of α is 35%. The low precision in the second estimate reflects the very low power available due to the low divergence in *An. gambiae*-*An. arabiensis *comparisons

One likely reason for the difference between *Anopheles *and *Drosophila *studies is the dearth of suitable outgroups for *An. gambiae *[e.g. [32]]. First, well-studied *Anopheles *species outside of the gambiae complex appear to be too distantly related to reliably infer the divergence between them. For example, the *An. stephensi *SRPN6 cDNA sequence [26] suggests that *K*_*S *_between with *An. stephensi *and *An. gambiae *is ~1.33 (± 0.157) substitutions per site when estimated by maximum likelihood [96], or 0.92 (± 0.117) by the method of Li [97]. Second, low divergence within the complex means there is little power to infer the substitution rate between them [6,32,33]. For example, across all 8 serpins appearing in the extensive survey of Cohuet *et al*. [6], there are only two fixed amino acid differences between *An. arabiensis *and *An. gambiae*. Similarly, the present study found no fixed differences at all in the same genes, probably because wider geographic sampling provided greater power to distinguish between polymorphisms and fixed differences. The stochastic errors involved here mean that estimates of substitution rate are likely to be wildly variable, reducing the power to estimate the fraction of adaptive substitutions using a McDonald-Kreitman framework (Figure 4).

In principle, other species from within the gambiae complex might be informative outgroups for *An. gambiae *and *An. arabiensis*: although divergence is small (1 to 5%; Figure 3a), this may be sufficient in other taxa, such as the human-chimp comparison [e.g. [98]]. However, unlike the human-chimp case, diversity in *Anopheles *is very high compared to humans (π_s_~3% in *An. gambiae*, ~2% in *An. arabiensis*, Figure 1, c.f. ~0.1% in humans, e.g. [99]), and this leads to two potential problems. First, a large proportion of apparent substitutions will in reality be polymorphisms [30,51], and although this effect is small enough to be negligible for pairs of species in which *K*_*S *_>> *π*_*S*_, it becomes a concern when comparing *An. gambiae *to *An. arabiensis*, for which *K*_*S *_<*π*_*S *_(Figure 3). However, as here, this effect can be accounted for using models which include the sample size and diversity, and thereby infer the 'true' number of substitutions [30,51]. Second, and potentially more serious, is the opportunity for extensive shared polymorphism. The McDonald-Kreitman framework uses information from current diversity (i.e. π_s _and π_a_) to infer whether some proportion of historic substitutions (*K*_*A*_) cannot be explained by purely neutral processes. This model implicitly assumes a time period when the two lineages were diverging from their common ancestor, during which selection (that is to be detected) was able to act. If there is extensive shared polymorphism between the species, for example if gene trees are rarely reciprocally monophyletic (as is the case with *An. gambiae *and *An. arabiensis*; see Figure 3b–d and e.g. [33]), then it is hard to see how the McDonald-Kreitman approach can ever be usefully applied. In other words, unlike the straight-forward cases where divergence is too high (the branch is too long) or divergence is too low (the branch is too short), for *An. gambiae *and *An. arabiensis*, in effect there is no branch at all (Figure 3b–d).

Unfortunately, for similar reasons, there are also serious concerns about the application of other selection-inference methods to the gambiae complex, such as the phylogenetic methods implemented in PAML [96] and HyPhy [100]. These methods use multiple sequences related by a gene (or species) tree to infer relative rates of synonymous and non-synonymous substitution, allowing variable rates at different sites or in different parts of the gene. Most phylogenetic methods assume there is no recombination within loci [but see OmegaMap, [101]], and simulation suggests false positives can reach >50% when 2*N*_*e*_*r *> 0.01 [102]. Because 2*N*_*e*_*r *(i.e: 2 × effective population size × recombination rate per codon per generation) in *An. gambiae *is likely to be of the order 0.01 – 0.1 or higher – primarily due to the large effective population size – such phylogenetic approaches cannot be applied reliably to within-species *Anopheles *data.

Additionally, where the McDonald-Kreitman framework assumes that between-species *K*_*A *_results from the joint action of selection and drift, and within-species *π*_*a *_results only from drift, the phylogenetic approaches (as they are most commonly applied) assume either that all amino acid variants have the same cause (i.e. *K*_*A *_and *π*_*a *_do not provide independent information about selection and drift) or that most differences are fixed between species, (i.e. *K*_*A *_>> *π*_*a*_, such that *π*_*a *_is negligible). Thus the phylogenetic and McDonald-Kreitman approaches constitute very different models that lend themselves to different datasets, and it is not clear that any single dataset can reasonably be analysed using both. Instead, to analyse a dataset that includes substantial within-species sampling using a phylogenetic approach, it may be more rational to fit a model which allows the relative rates of synonymous and non-synonymous substitution to differ between within- and between-species branches [e.g. [103]]. However, in the case of the *gambiae *complex, the low power associated with the low inter-species divergence will then be encountered again.

If McDonald-Kreitman tests have relatively low power within the gambiae complex, and phylogenetic methods cannot easily be applied to within-species data, how can selection be inferred from *Anopheles *population genetic data? One possibility is to use approaches based solely on within-population diversity to identify recent selective sweeps or regions of elevated polymorphism, and this will work for some loci [e.g. TEP1, [92]], though the possibility of introgression, and/or chromosomal inversions that might affect the distribution of diversity, should then be taken into account. Nevertheless, at present it seems the best options for outgroup-based analyses are *An. merus *and/or *An. melas*, and both the data presented here (Figure 3) and previous studies [e.g. [33,66]] suggest that their divergence from the *An. gambiae*/*An. arabiensis *clade should be sufficient in the case of genes evolving under very strong selection. However, the recently approved genome sequences from 13 more species of *Anopheles *mosquitoes [82] may hold the solution, and particularly the complete genome sequence of *Anopheles sundaicus *(also subgenus *Cellia*, *Pyretophorous *series).

## Competing interests

The authors declare that they have no competing interests.

## Authors' contributions

DJO selected the loci, designed the primers, and performed all PCR, sequencing and analysis. JJW developed the likelihood-based methods for inferring the proportion of adaptive substitutions, and provided statistical support. TJL conceived the project, and all authors contributed to the final manuscript.

## Supplementary Material

Additional file 1 **PCR primers**. Locus names, identifiers, genomic locations and PCR primer sequences. Click here for file

Additional file 2 **Correlations in genetic diversity between species**. The correlation in genetic diversity for loci sampled from *Anopheles gambiae *and *Anopheles arabiensis*. Click here for file

Additional file 3 **Genetic diversity and differentiation**. Sheet 1: genetic diversity at synonymous and non-synonymous sites, and Tajima's D statistic for synonymous sites, for all loci and populations. Sheet 2: genetic differentiation (Fst, Kst and Snn) between populations for all loci. Click here for file

Additional file 4 **McDonald-Kreitman data**. Sample sizes, analysed gene lengths, polymorphisms and fixed differences for each locus, for each pair of species Click here for file
