# Supplementary material for: Inferring selection in the Anopheles gambiae species complex: an example from immune-related serine protease inhibitors
Source: Malar J. 2009 Jun 4;8:117. doi: 10.1186/1475-2875-8-117 (PMC2698913; doi:10.1186/1475-2875-8-117)
Supplement: Additional file 1 — PCR primers. Locus names, identifiers, genomic locations and PCR primer sequences. [file 1475-2875-8-117-S1.doc]

| NAME | Identifier | Gene location | Forward | Reverse |
| --- | --- | --- | --- | --- |
| SRPN1 | AGAP006909 | 2L:39892128-39893864 | TGAATTCGATATTGGCACAAA | AAGCGTCCCCTTTTCGTT |
| SRPN2 | AGAP006911 | 2L:39897002-39899744 | GACACGAGTTTCGGCAATG | AGCGATGCGTTTTGTGAGAAGA |
| SRPN3 | AGAP006910 | 2L:39895229-39896338 | GGCGCTACTGTACGAAGGTT | AATGAACTTGACCGGTTGGT |
| SRPN4C | AGAP009670 | 3R:38145527-38154288 | GCACAACCAGACGCATGGTC | GAACAAGGGCAGTCTGGTCGT |
| SRPN5 | AGAP009221 | 3R:28858000-28859778 | CTTGAGGAGCCGATCGAA | GATCGAATACTGCCCCGTAA |
| SRPN6 | AGAP009212 | 3R:28811997-28818217 | GAATATCGGCCCGTAGAACA | GTCACAGGTAATCCGTGTCG |
| SRPN7 | AGAP007693 | 2L:49090665-49091915 | GTTCCGTTTTCGTCCACCT | TGGTCCCGATCTGAGCTTTGG |
| SRPN8 | AGAP003194 | 2R:33744972-33746720 | AAATTCGGTCGCTTCACTTG | CTGCAGCCGATGAACTTTG |
| SRPN9 | AGAP003139 | 2R:33148444-33154607 | CCGTCTTCTCGATGCTGAAC | CAAGGGCGAATCGATCTTTA |
| SRPN10 | AGAP005246 | 2L:12996143-13001508 | GCAGATCCTCGTTGAGATCG | AAACATGCGGGAGAGAATGT |
| SRPN11 | AGAP001377 | 2R:4017728-4019706 | AGCACCATGCAACGGTAATC | GTTTCCGCTTTGAACTCTGG |
| SRPN12 | AGAP001375 | 2R:4010431-4012512 | ATCGTTCCTAACCGGAGACA | TCGACGTACGGGATGTCCGT |
| SRPN14 | AGAP007692 | 2L:49084812-49086463 | CTGCAGAACAGCGTAGCGATG | AGGTGACCGATGAACAGGAG |
| SRPN16 | AGAP009213 | 3R:28824548-28826209 | GCAAATTGATGGACGGTAGC | CTGAATGGTGGCTTCACGTA |
| SRPN17 | AGAP001376 | 2R:4015617-4016537 | CACGTCTACTTATGGCGAGCGT | CAGCGGATACTCGTGTATCTTGTC |
| SRPN18 | AGAP007691 | 2L:49086842-49088278 | CGGTGCTGCAGTACAGGAA | AAGTTGCCCACTTGCAACA |
| Control1 | AGAP006906 | 2L:39852471-39854636 | CTGCTAGCCTCCGAGGAATC | AGTCCGCGAAACTCCAGATA |
| Control2 | AGAP006904 | 2L:39831595-39836700 | CTCTACCTCTCACAGTTTGGCTA | CCCTTGAAGGCGTAGGTGC |
| Control3 | AGAP006918 | 2L:39995907-39997095 | CCCTAGTTGAGCCAGTGGAA | GACGGAGGAAGAACTTACG |
| Control4 | AGAP009673 | 3R:38248845-38249780 | ACACAGCCACCCCGATGT | CCAGCACAAAGACGGAAAAT |
| Control5 | ENSANGG8091 | 3R:28965847-28968579 | GAAGCATTTCAAGCGCTGTTCAC | AACACTCCCGGATACTGTCG |
| Control6 | AGAP009207 | 3R:28697030-28708787 | GTCCGCCGGATCGTAGTA | TTGTGATTTCGGTCTTGCAC |
| Control7 | AGAP007712 | 2L:49181235-49190516 | TGAATTTATCATGACTGAGAAGCA | TGTCCGACTTTTTGAATTTGT |
| Control8 | AGAP003205 | 2R:33825401-33827998 | CGTATCGTCCGGAGTCATCT | TCTTCTGCAACGTGATCGTC |
| Control9 | AGAP003143 | 2R:33211906-33213476 | TTTGGACCCTTTCAAAATCG | CGACAGTGGATGGTGATGTC |
| Control10 | AGAP005247 | 2L:13062962-13067750 | CCGCTAGTCAGCTCCTGCT | CCCGATCGTTTTCATTCTTC |
| Control11 | AGAP001384 | 2R:4098545-4103634 | GTTGCGCAGCAGATCCTT | GACTTCGAGCGGATCAAGA |
| Control12 | AGAP001371 | 2R:3885127-3885956 | TGAACTGCTTTCCGATTCCT | GATGAACTGGTGAGCTCGAT |
| Control14 | AGAP007713 | 2L:49196817-49198177 | CAATGCCAACAAAGACAAGG | CGGAAGAATATCGTCGGCTA |
| Control16 | AGAP900209 | 3R:28746535-28747425 | AACGCACATCACACAGTTGG | TGCACTAAAACGCCAACAAG |
| Control17 | AGAP001388 | 2R:4120810-4122586 | CACCGACCTTCGAATCTTTC | CCACCCTCCTTCTTTTCCTT |
| Control18 | AGAP007717 | 2L:49212258-49224889 | CTTTGGGGCACTGTTGCTAT | GTCCTTTGGGGCACTGTTGC |

Supplementary Table S1: PCR primers
