# Supplementary material for: Inferring selection in the Anopheles gambiae species complex: an example from immune-related serine protease inhibitors
Source: Malar J. 2009 Jun 4;8:117. doi: 10.1186/1475-2875-8-117 (PMC2698913; doi:10.1186/1475-2875-8-117)
Supplement: Additional file 2 — Correlations in genetic diversity between species. The correlation in genetic diversity for loci sampled from Anopheles gambiae and Anopheles arabiensis. [file 1475-2875-8-117-S2.doc]

Supplementary Figure S1: Correlations in genetic diversity between species
